# A Novel Polymer Inclusion Membrane-Based Green Optical Sensor for Selective Determination of Iron: Design, Characterization, and Analytical Applications

**DOI:** 10.3390/polym15204082

**Published:** 2023-10-14

**Authors:** Lorena Sánchez-Ponce, María José Casanueva-Marenco, Margarita Díaz-de-Alba, María Dolores Galindo-Riaño, María Dolores Granado-Castro

**Affiliations:** Department of Analytical Chemistry, Institute of Biomolecules (INBIO), Faculty of Sciences, International Campus of Excellence of the Sea (CEI-MAR), University of Cadiz, Campus Rio San Pedro, Puerto Real, 11510 Cadiz, Spain; lorena.sanchezponce@alum.uca.es (L.S.-P.); mariajose.casanueva@uca.es (M.J.C.-M.); dolores.galindo@uca.es (M.D.G.-R.); dolores.granado@uca.es (M.D.G.-C.)

**Keywords:** iron determination, iron speciation, optical sensor, polymer inclusion membrane, picolinaldehyde salicyloylhydrazone

## Abstract

The design, characterization, and analytical application of a green optical sensor for the selective determination of Fe(II) ions is proposed. The sensor is based on the immobilization of the chromogenic reagent picolinaldehyde salicyloylhydrazone (SHPA) within a polymer inclusion membrane. To reduce solvent usage, the reagent was synthesized using a green mechanochemical procedure. The components for sensor preparation were optimized with a sequential simplex method and the optimal composition was found to be 0.59 g cellulose triacetate (base polymer), 0.04 g SHPA (chemosensor reagent), 4.9 mL dibutyl phthalate (plasticizer), and 38 mL dichloromethane (solvent). The conditions of iron analysis were also optimized resulting in pH 6 for aqueous solution, 90 min exposure time and 10 min short-term stability. The optical sensor showed a linear range from the limit of detection (0.48 µmol L^−1^) to 54 µmol L^−1^ Fe(II). The precision of the method was found to be 1.44% and 1.19% for 17.9 and 45 µmol L^−1^ Fe(II), respectively. The characteristics of the sensor allowed the design of a Fe(II)/Fe(III) speciation scheme. The methodology was successfully applied to the determination of iron in food preservatives, food additives, and dietary supplement. Additionally, the Fe speciation scheme was successfully applied to an agricultural fertilizer.

## 1. Introduction

In recent years, there has been a growing interest in the development of green analytical approaches to the analysis of metal ions in real samples, which involve lower reagent consumption, minimal waste generation, and/or low hazardous waste in order to ensure safety and preserve the environment. 

In this sense, a chemical sensor can be considered as a green analytical approach to the determination of chemical species. It is defined as a device that reacts to a specific analyte producing a signal, which can be used for its qualitative or quantitative determination [1]. Chemical sensors can be classified into electrochemical, optical, mass, magnetic, and thermal depending on the traducer type [2].

In recent years, the development of optical chemical sensors (also known as optodes) has increased due to their characteristics. They can be applied to analyze different chemical species and they offer some advantages compared to conventional methods: simple use, high selectivity and sensitivity, low cost of operation, fast response, and high chemical and physical versatility [3,4,5].

Optical chemical sensors can be based on different optical principles such as absorbance, reflectance, luminescence, or fluorescence, covering a wide range of the spectrum from ultraviolet to near infrared, using the simplicity of photometric measurements [6]. 

The immobilization of a reagent on the solid substrate of the sensor followed by its binding with the analyte produces a change in the optical properties of the solid phase [4]. The change in these properties can correlate with the concentration of the analyte, allowing its detection and/or quantification [4]. 

Polymer inclusion membranes (PIMs) have been widely used as sensing components in optodes [6,7,8,9]. They have a high interfacial surface, exhibit high selectivity, and provide good efficiencies and ease of operation. Their preparation and application require the use of small amounts of reagents, and they are considered versatile instruments since different membrane compositions can be selected. The most important advantages of PIMs are their low cost of processing [10] and the possibility to perform in-situ metal analysis with different techniques such as UV-Vis spectroscopic technique [11]. PIMs are flexible films that can be prepared using a base polymer, such as cellulose triacetate (CTA) or poly(vinyl chloride) (PVC), a reagent (to facilitate the binding with the analyte), and a plasticizer (also defined as modifier)—such as 2-nitrophenyl octyl ether (NPOE), tributyl phosphate (TBP), or dibutyl phthalate (DBP)—to provide elasticity and neutralize the polar groups of the polymer, reducing intermolecular forces due to their different chemical structures, dielectric constants, and viscosities [7,12]. Furthermore, PIMs can also contain different additives, which can act as anionic counter-ions or as extractant agents to facilitate the diffusion of the target species from the solution to the sensor membrane. Some examples of additives are potassium tetrakis(4-chlorophenyl)borate or sodium tetraphenylborate as anionic counter-ions [13], and Titron X100, N-methyl-N,N,N-trioctylammonium chloride (Aliquat-336), or di-(2-ethylhexyl)phosphoric acid (D2EHPA) as extractant agents [3,14,15]. 

PIMs are usually prepared by dissolving all membrane components in a small volume of a volatile solvent and casting the solution onto a specific surface. The solvents most frequently found in the literature are dichloromethane (DCM) and tetrahydrofuran (THF), both used in minimal amounts [7,10]. The solvent is evaporated, and a thin polymeric film is formed.

The selection of the membrane components is an important step. The polymer matrix provides selectivity, flexibility, and chemical and mechanical stability to the optode. According to the literature, PVC and CTA are the polymers that provide the best results in the design of optical sensors [12,15,16]. While CTA is a biodegradable polar polymer with very good optical properties and capable of forming highly oriented hydrogen bonds, PVC is capable of creating intermolecular interactions because it is a relatively inexpensive polar polymer [3,15,17,18]. 

When PIMs are used as optical sensors to determine metal ions, the addition of reagents with chromophore groups is of great importance. Different reagents with chromophore groups have been used in the literature, such as dithizone [19], xylenol orange [20], chromeazurol S [21], 4-(2-pyridylazo)resorcinol (PAR) [15,22], 1-(2-pyridylazo)-2-naphtol (PAN) [23], 4-(2-thiazolylazo) resorcinol (TAR) [24], Br-PADAP [25], 2-amino-1-cyclopentene-1-dithiocarboxylic acid (ACDA) [26], 1-nitroso-2-naphthol (NN) [27], and pyrocatechol violet (PV) [28]. However, most of these reagents provide low selectivity for metal ions [29]. The Schiff bases containing =C=N–N= groups (hydrazones) are considered very interesting tools in the design of PIMs due to their metal complexing and chromogenic properties, when optical measurements are required [30,31,32]. 

PIMs as membrane based optical sensors have been used to quantify different metals by spectrophotometry, spectrofluorometry, or color-intensity measurements, among others (e.g., Al(III) in lake and river water samples [3,13,14], Cu(II) in river water and isotonic seawater samples [13,29], Co(II) in tap water, seawater, and wastewater samples [6,27], Fe(III) in isotonic seawaters [29], Hg(II) in surface and groundwater samples [15,18], Pb(II) in plastic toys and tap water samples [33], Tl(I) in aqueous solution [17] and Zn(II) in pharmaceuticals samples, vitamin-mineral drinks, food supplements and foot health care products [12,29]).

The analysis of Fe is an important issue because it is an essential element involved in many biochemical and physiological processes of living systems. Both excess and deficiency of Fe in organisms can lead to health problems [34]. Iron is generally present in two oxidation states as Fe(II) (ferrous) and Fe(III) (ferric), which play an important role in the functioning of respiratory enzymes [35]. It is well known that the chemical forms of Fe influence its bioavailability and its physical-chemical and toxicological properties. In fact, biological systems mainly use Fe(II) because it is easier to be assimilated than Fe(III) [36]. In open oceans, Fe(II) is preferred as a nutrient by phytoplankton and acts as a limiting factor in their growth [37]. Therefore, iron speciation is essential in environmental and biological studies [38]. There are many well-known methods in the literature to determine iron, applying different techniques such as inductively coupled plasma-optical emission spectroscopy (ICP-OES), high-performance liquid chromatography (HPLC), or stripping voltammetry, among others [39,40,41]. These methods are highly sensitive and selective, but they are expensive and require complex sample preparation, a trained operator, and are unsuitable for field applications. In contrast, the use of PIMs followed by a colorimetric detection has advantages such as simplicity and low cost. This methodology, based on the color change of the PIM by complexation of the metal ions with a suitable chelating agent, is of great interest in the case of Fe(II), which can form stable colored complexes with organic reagents. Optical sensors for Fe(II) are still rare in the literature [29,42,43,44,45,46,47,48].

**Figure 1:**
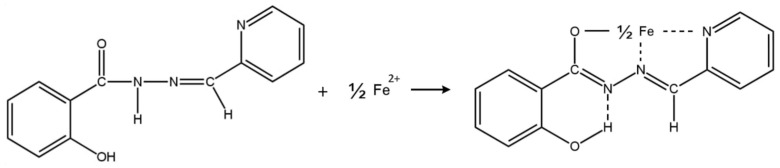
Mechanism of the sensing phenomena.

This paper is focused on the development of an easy colorimetric method for the determination of Fe(II) using a polymer inclusion membrane as an optical sensor. The potential application of the optode for the speciation of Fe(II)/Fe(III) is studied. The hydrazone picolinaldehyde salicyloylhydrazone (SHPA, molecular formula: C_13_H_11_N_3_O_2_, molecular weight: 241) was immobilised on the membrane as a chromogenic reagent due to its interesting properties as a metal ion complexing agent. SHPA has a solubility in water, methanol, ethanol, chloroform, nitrobenzene, benzene, and amyl alcohol of 0.2, 1.1, 1.0, 0.04, 0.7, 0.5 and 0.9 g L^−1^, respectively. This ligand shows a maximum absorption at 300 nm in different solvents as water, amyl alcohol, or chloroform, at 305 nm in ethanol, and at 325 in acetone. The average pK values found are 3.5 ± 0.1 (associated with the protonation of the pyridine nitrogen atom) and 9.2 ± 0.1 (associated with the hydroxyl group) [49]. This reagent forms chromogenic complexes with different metal ions such as Al(III) (blue fluorescence), Fe(II) (green complex) [50], or Bi(III), Co(II), Cu(II), Pd(II), Ti(IV), V(V), and Zn(II) as yellow complexes [51,52,53]. This chromogenic group –N=C–C=N–NH–CO of the SHPA can act as a tridentate ligand, which interacts with Fe(II), forming an octahedral complex with four five-membered rings [54] (Figure 1).

The green-colored complex of SHPA with Fe(II) allows selective spectrophotometric measurements. Thus, the optimization of the PIM components, its characterization, and the analytical application of the method will be discussed in detail.

## 2. Materials and Methods

### 2.1. Reagents and Solutions

All the reagents and solvents were of analytical or Suprapur grade, and all the solutions were prepared using ultra-high-quality water. Stock aqueous solutions of Fe(II) were prepared using either a Fe(III) ICP standard solution of 1000 mg L^−1^ in 0.05 mol L^−1^ HNO_3_ (Certipur, Merck, Darmstadt, Germany) in the presence of 0.0289 mol L^−1^ ascorbic acid (for analysis grade, Panreac, Castellar del Vallès, Barcelona, Spain), or from Mohr’s salt ((NH_4_)_2_Fe(SO_4_)_2_·6H_2_O, Merck, Darmstadt, Germany). Acetic acid/acetate buffer solutions (4 mol L^−1^, pH 3.5–5.5) were prepared using acetic acid (analytical grade, 96%, Merck, Darmstadt, Germany) and sodium hydroxide (analytical grade, Panreac, Castellar del Vallès, Barcelona, Spain). Britton-Robinson buffer solutions (3 mol L^−1^, pH 5–7.5) were prepared using ortho-phosphoric acid (analytical grade, 85%, Merck, Darmstadt, Germany), boric acid (analytical grade, Merck, Darmstadt, Germany), and potassium chloride (analytical grade, Panreac, Castellar del Vallès, Barcelona, Spain). Ammonium-chloride buffer solutions (3 mol L^−1^, pH 8–10) were prepared using ammonia solution (25%) and hydrochloric acid (37%) (Merck, Darmstadt, Germany). The organic reagent (picolinaldehyde salicyloylhydrazone, SHPA) was synthesized by the reaction of salicyloyl hydrazide (Sigma-Aldrich, St. Louis, MO, USA) and 2-pyridinecarboxaldehyde (Sigma-Aldrich, St. Louis, MO, USA), using distilled ethanol (Scharlab, Spain). Cellulose triacetate (CTA) (Sigma-Aldrich, St. Louis, MO, USA) and poly(vinyl chloride) (PVC) (Sigma-Aldrich, St. Louis, MO, USA) as base polymers, dibutyl phthalate (DBP) (Merck, Darmstadt, Germany) and tributyl phosphate (TBP) (Sigma-Aldrich, St. Louis, MO, USA) as plasticizers, and dichloromethane (DCM) (Panreac, Castellar del Vallès, Barcelona, Spain) and tetrahydrofuran (THF) (Panreac, Castellar del Vallès, Barcelona, Spain) as volatile solvents were used to prepare the PIM. 

Interferences were studied by using Ag(I), Bi(III), Cd(II), Co(II), Cr(III), Cu(II), Fe(III), Mg(II), Ni(II), Pb(II), and Zn(II) ICP standard solutions of 1000 mg L^−1^ in 0.05 mol L^−1^ HNO_3_ (Certipur, Merck, Germany), sodium chloride (Panreac, Castellar del Vallés, Barcelona, Spain), sodium fluoride (Merck, Darmstadt, Germany), potassium chloride (Panreac, Castellar del Vallés, Barcelona, Spain), and thioglycolic acid (Panreac, Castellar del Vallés, Barcelona, Spain).

To compare the results obtained by the optical sensor with the results obtained by the ortho-phenanthroline method, 1,10-phenanthroline-monohydrate, hydroxyl-ammonium chloride, Mohr’s salt ((NH_4_)_2_Fe(SO_4_)_2_·6H_2_O), and ammonium acetate (Merck, Darmstadt, Germany) were used.

### 2.2. Instrumentation

Water was ultra-purified by reverse osmosis with an Autwomatic (Water type II) system followed by ion exchange with an 18.2 MΩ cm deionized Ultramatic Plus system (Wasserlab, Barbatáin, Navarra, Spain). To determine the pH of the solutions, a Basic 20 pH-meter with a 50_10T combined glass-Ag/AgCl electrode (Crison, Barcelona, Spain) was used. An HS 501 D shaking platform (Ika, Labortechnik, Staufen, Germany) was used to shake the samples. 

The organic reagent (SHPA) was synthesized using a greener process than the one usually used by other authors for hydrazones, based on the condensation of the reagents under refluxing conditions in ethanol medium [50,55,56]. Thus, the synthesis was carried out by a mechanochemical process mixing salicyloyl hydrazide and 2-pyridinecarboxaldehyde in a Retsch MM2 mixer mill with a 10-mm single ball in a ZrO_2_ jar (Retsch GmbH, Haan, Germany). 

The synthesis of the PIM was carried out with a Q700 high-energy ultrasound generator (Qsonica Sonicators, Newtown, CT, USA), and the solvent was allowed to evaporate inside a fume hood (Flowtronic, Romero S.A., Torrejón de Ardoz, Madrid, Spain). The sensor was placed onto a handmade polyester film support (thickness; 25 µm, Mylar^®^, Dupont, Hopewell, VA, USA) for UV-Vis measurements), and a UV-Vis spectrophotometer (Jasco, Hachioji, Tokio, Japan) controlled by Spectra Manager^TM^ software version 2.07.02 (Jasco, Hachioji, Tokio, Japan) was used to determine the absorbance values. These values were then exported to Microsoft Excel 2016 (Microsoft Corporation, Redmond, WA, USA) for data processing.

### 2.3. Mechanochemical Synthesis of SHPA and PIM Preparation

The organic reagent (SHPA) was synthesized by a green mechanochemical process using equimolar amounts of salicyloyl hydrazide and 2-pyridinecarboxaldehyde and using 75% less than the usual amount of distilled ethanol in a mixer mill at 60 Hz.

A sequential simplex by means of the software SOVA 1.0 (©Luboš Svoboda, 2011) was used to optimize the amount of PIM components for Fe(II) determination. Using the simplex, the effects and interactions of several components (such as amounts of CTA and SHPA or volumes of DBP and DCM) on the response of the optical sensor were evaluated. The solution casting method was used to prepare the PIM. Thus, different amounts and volumes of the components were dissolved using an ultrasound generator (8 min of sonication pulses at 2 s intervals) to obtain a homogeneous mixture. The mixture was then placed in an 11.7 cm diameter glass Petri dish, which was covered with a piece of filter paper to allow the DCM to evaporate into a dark fume hood for a curing time at a control room temperature of 20 °C. The resultant PIM was sliced into portions of 2.37 cm × 0.98 cm and stored, in the absence of light, inside a desiccator until used as an optical sensor. 

### 2.4. General Analytical Procedure for Optical Fe(II) Ion Sensing

Batch experiments were carried out in duplicate, and consisted in letting the optical sensor equilibrate with the metal solution and the subsequent measurement of the absorbance of the complex formed between Fe(II) and the Schiff base (SHPA). The sensor was exposed to 20 mL of metal (or blank) solution under certain conditions for each experiment (PIM composition: amounts of CTA (g), SHPA (g), DBP (mL), and DCM (mL); conditions of the metal solution: metal concentration and pH (adjusted with buffer); and time of exposure) in 100 mL polypropylene containers. The containers were shaken at 300 rpm at a controlled temperature of 20 °C during the time of exposure. 

The sensor was then taken out from the container, rinsed with ultra-purified water, dried with absorbent paper and placed onto a polyester film (4.75 cm × 0.98 cm size, used as a support for the PIM inside the quartz cuvette). The sensor was kept for a while in a desiccator protected from light.

A UV-Vis spectrophotometer was finally used to evaluate the absorbance of the optical sensor at either 389 or 645 nm (wavelengths of maximum absorption of the Fe(II)–SHPA complex). 

**Figure A1:**
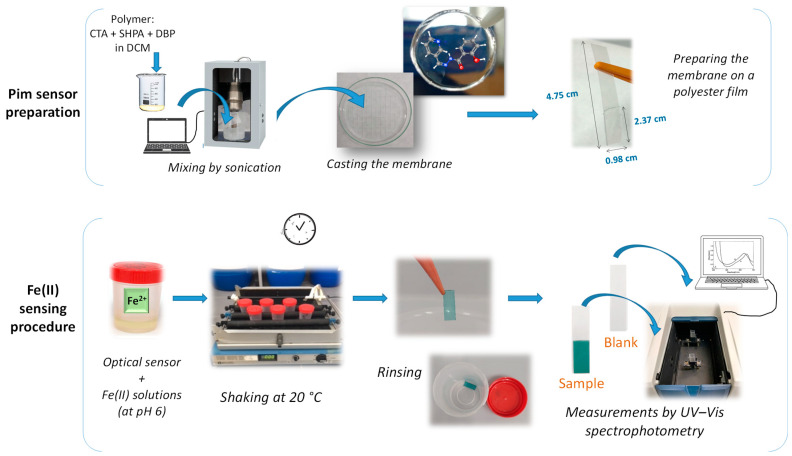
Experimental setup of the proposed method.

The experimental setup is shown in Figure A1 in Appendix A.

### 2.5. Real Samples

Real samples were analyzed using the proposed optical sensor under optimal conditions. The real samples analyzed in this paper were black olive brine from Karina, s.l. (Spain), black olive brine from Ifa Eliges (Spain), food additive E579, and Tardyferon (pharmaceutical compound, Pierre Fabre Iberica, Spain). The black olive brine samples were directly analyzed with the proposed optical sensor under optimal conditions. The food additive sample was prepared by dissolving 0.2 g of additive in 1 L of ultra-purified water, which was then four-times diluted. As for the Tardyferon sample, an 80 mg tablet was ground and dissolved in 2 L of ultra-purified water.

To evaluate the accuracy and reliability of the suggested approach to the determination of Fe(II) ions, the results were compared with those obtained from the ortho-phenanthroline method [57]. For that purpose, a solution was prepared for each real sample (2.5 mL of black olive brine from Karina; 10 mL of black olive brine from Ifa Eliges; 2 mL of food additive E579 solution; 2 mL of Tardyferon solution) with 5·10^−4^ mol L^−1^ 1,10-phenanthroline-monohydrate, 2.35 mol L^−1^ ammonium acetate buffer solution, and 0.48 mol L^−1^ hydrochloric acid in 50 mL. After 20 min the solutions were measured by spectrophotometry at 510 nm. 

For the speciation of Fe(II)/Fe(III) analysis, a fertilizer (6% *w*/*w* of water-soluble Fe content Transchel Supra, BC. Fertilis, S.L., Spain) was used. A solution was prepared by dissolving 0.02 g of fertilizer in 1 L of ultra-purified water. Different aliquots were analyzed with the designed optical sensor under optimal conditions, with and without the addition of 0.0289 mol L^−1^ ascorbic acid for the respective determination of total Fe (Fe(II) + Fe(III)) and Fe(II). 

## 3. Results and Discussion

### 3.1. Previous Studies

The polymers selected for the previous studies were poly(vinyl chloride) (PVC) and cellulose triacetate (CTA). Most PIMs that use CTA as polymer use chloroform as the volatile solvent; however in this study dichloromethane (DCM) has been used, which is greener than chloroform.

**Figure A2:**
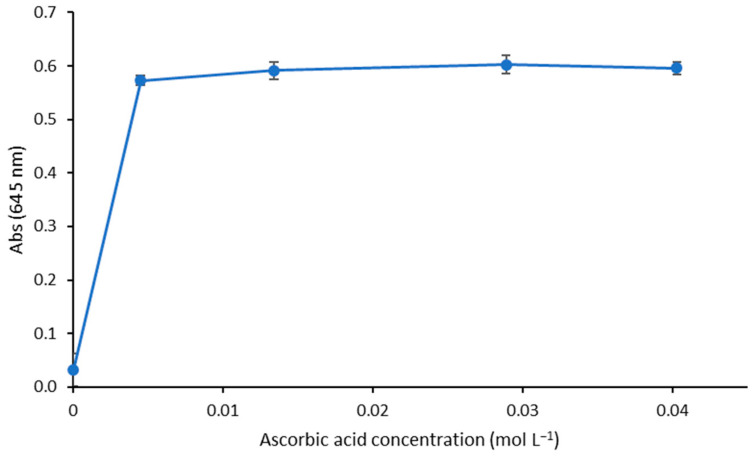
Effect of the ascorbic acid concentration (0, 0.0045, 0.0134, 0.0289, and 0.0403 mol L^−1^) on the sensor response exposed to 45 µmol L^−1^ Fe(II).

**Figure A3:**
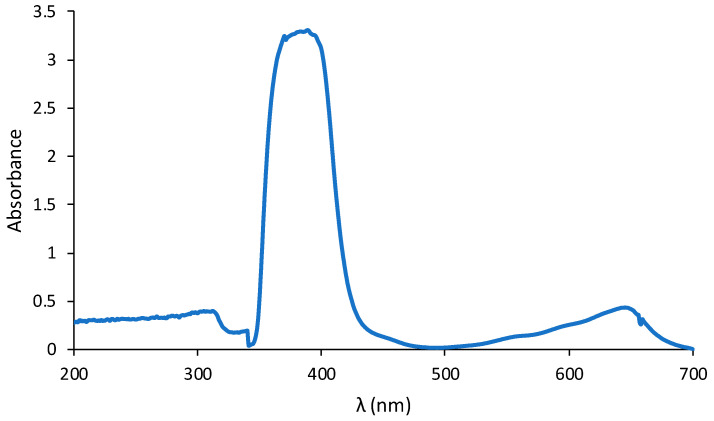
UV-Vis absorbance spectrum of the optical sensor with CTA, DBP, DCM, and SHPA for 90 µmol L^−1^ Fe(II).

Based on the literature, and in order to establish the type of polymer, plasticizer, solvent, and potential additive to be used in the PIM preparation, different membranes with SHPA as colorimetric reagent were prepared and exposed to a 20 mL metal solution containing 90 µmol L^−1^ Fe(II), buffered by a 0.337 mol L^−1^ acetic acid/sodium acetate solution (at pH 4.6) or Britton-Robinson solution (at pH 2.5 and 9). An excess of ascorbic acid was added to the Fe(III) stock solution (see the optimization of the ascorbic acid concentration in Figure A2 in Appendix A). The containers were shaken at 300 rpm at a controlled temperature of 20 °C for 90 min. The absorbance of the optical sensor was then evaluated. As can be seen from the UV-Vis absorbance spectrum (Figure A3 in Appendix A), the maximum absorbance of the Fe(II)–SHPA complex was found in two different wavelengths: 389 and 645 nm. The former allows a more sensitive determination of Fe(II) concentration, but it is subject to interferences because other metal ion complexes show a peak at this wavelength [50,51,52,53]. The latter offers lower sensitivity but higher selectivity. For this reason, in this work the sensor response was evaluated at 645 nm.

Table 1 shows the different compositions of the synthesized PIM and the results obtained after metal exposure. The highest response was obtained using CTA, DBP, and DCM at pH 4.6. 

### 3.2. Optimization of the PIM Composition 

To select the optimal amounts of CTA, DBP, DCM, and SHPA for the synthesis of the optical sensor, a sequential simplex optimization was carried out. The sensor was prepared as defined in Section 2.3 and exposed to a metal solution containing 90 µmol L^−1^ Fe(II), buffered by a 0.337 mol L^−1^ acetic acid/sodium acetate solution at pH 4.6, for 60 min. The initial simplex was defined by five sensing experiments (five vertices) because of the number of variables to optimize. Based on the absorbance values obtained from the initial simplex, the following vertex was established. The conditions and responses (absorbance values at 645 nm) of the experiments are shown in Table 2.

According to the simplex method, the variances of each simplex and of the method were compared in order to stop the simplex, using the F-value:

(1) $$F_{calculated}=\sigma_{simplex}^{2}/\sigma_{exp}^{2}$$

where *σ*^2^*_simplex_* is the variance of the simplex and *σ*^2^*_exp_* is the variance of four replicates of one experiment. When the *F_calculated_* was lower than the *F_tabulated_* (after 17 experiments), the simplex was stopped. Taking into account the responses and the standard deviations obtained in each experiment, as well as the reagents amounts/volumes used in the synthesis of the PIM, the conditions of the experiment number 14 were selected as the optimal: 0.59 g CTA, 0.04 g SHPA, 4.9 mL DBP, and 38 mL DCM. This sensor composition was used in the following experiments. 

### 3.3. Lifetime of the Optical Sensor

The lifetime of the optical sensor was studied in order to acknowledge how long the sensor could be used after its preparation. For that purpose, a sensor was synthesized under the optimal conditions described in Section 3.2. At 6, 12, 24, and 72 h after the synthesis, an absorbance at 350 nm (due to the Schiff base) was observed. The findings from three replicates revealed that the absorbance remained constant for 72 h. There were no significant differences between the results obtained at 6 and 72 h according to a *t*-test (95% confidence). Therefore, the PIM could be used up to 3 days after synthesis.

### 3.4. Effect of pH and Buffer Concentration on the Response of Sensor

**Figure 2:**
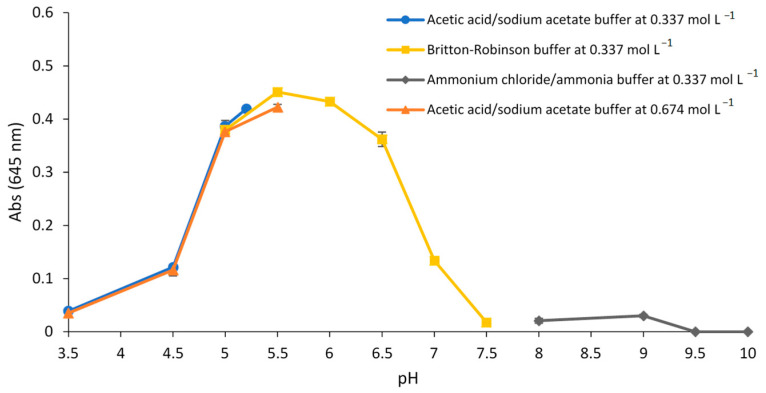
Effect of the pH and type of buffer solution on the sensor response exposed to 90 µmol L^−1^ Fe(II).

The influence of the pH value and the type of buffer on the sensor response (absorbance value at 645 nm due to the Fe(II)–SHPA complex) was studied. For this, acetic acid/sodium acetate (pH 3.5, 4.5, 5, 5.2 and 5.5), Britton-Robinson (pH 5, 5.5, 6, 6.5, 7 and 7.5), and ammonium chloride/ammonia (pH 8, 9, 9.5 and 10) buffers at a concentration of 0.337 mol L^−1^ were tested. The metal solutions contained 90 µmol L^−1^ Fe(II) and the response of the sensor was evaluated after 60 min of exposure. In addition, the influence of the acetic acid/sodium acetate buffer concentration on the response was evaluated and the experiments were performed using a concentration of 0.674 mol L^−1^. The results (Figure 2) indicated that the best responses were obtained at pH 5.5 and pH 6, and the optimum concentration of buffer was 0.337 mol L^−1^.

**Figure 3:**
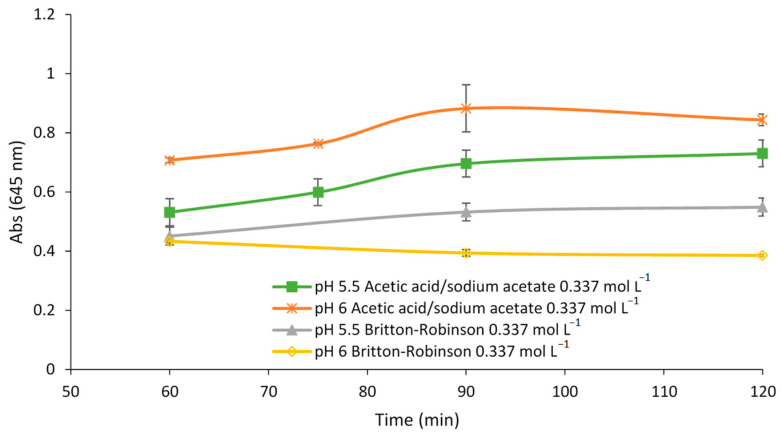
Response of the sensor to 90 µmol L^−1^ Fe(II) at different times of exposure, pH values, type of buffer solutions, and concentrations of buffer.

To delimit the optimal pH solution and select the best buffer, the sensor was exposed to a metal solution containing 90 µmol L^−1^ Fe(II) at different exposure times (60, 75, 90, and 120 min), buffered with acetic acid/sodium acetate or Britton-Robinson solution at pH 5.5 and 6. As can be seen in Figure 3, the highest absorbance value was obtained at pH 6 with acetic acid/sodium acetate solution at any studied exposure time. 

### 3.5. Response Time

**Figure 4:**
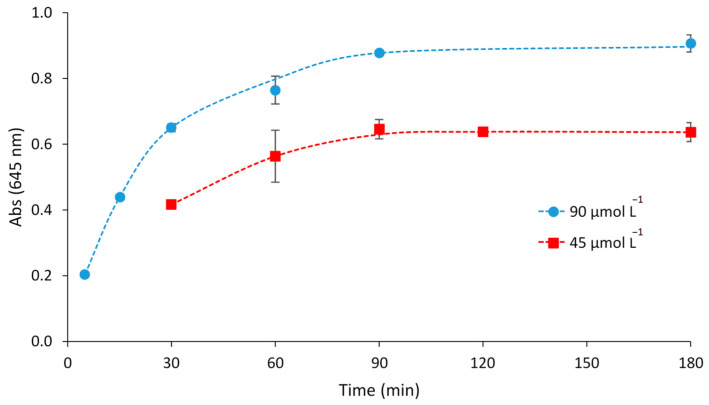
Response time of the optical sensor at different concentrations of Fe(II) (0.337 mol L^−1^ acetic acid/sodium acetate buffer, pH 6, *n* = 2).

The optical sensor was exposed to a metal solution containing 90 or 45 µmol L^−1^ Fe(II) buffered with 0.337 mol L^−1^ acetic acid/sodium acetate solution at pH 6, for different exposure times (5, 15, 30, 60, 90, 180 min) at 300 rpm. The results of this study (Figure 4) revealed that the adequate time of exposure to obtain a maximum and constant signal was 90 min. 

### 3.6. Curing Time of the Optical Sensor

**Figure 5:**
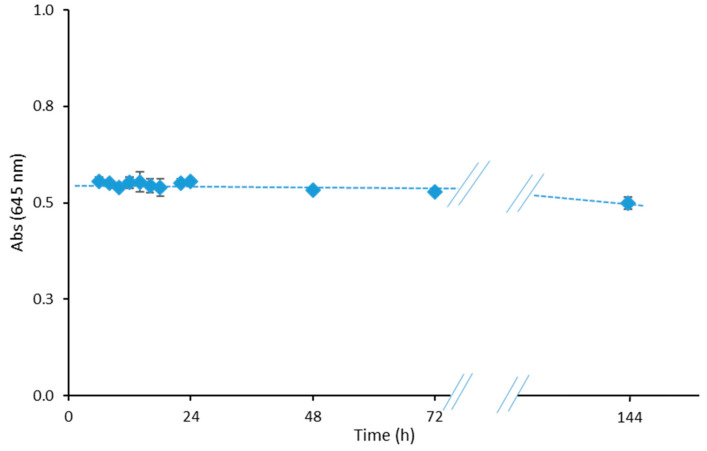
Effect of different curing times on the sensor response exposed to 45 µmol L^−1^ Fe(II).

The curing time of the PIM is the time lag between the synthesis of the PIM and its exposure to the metal solution without changes in the sensor response. To study the curing time of the sensor, the response was evaluated after exposure to 45 µmol L^−1^ Fe(II) at optimal conditions after 6, 8, 10, 12, 14, 16, 18, 20, 22, 24, 48, 72, and 144 h of curing time. The effect of different times on the sensor response is shown in Figure 5. The sensor response did not vary in the first 72 h, and the slight decrease for longer periods is not significant.

### 3.7. Short-Term Stability

To assess the short-term stability of the sensor, the absorbance of the optical sensor at 645 nm was measured at different times after the exposure to the metal solution within a 300 min period. For that purpose, the sensor was exposed to 45 µmol L^−1^ Fe(II) at optimal conditions. The results showed a constant signal in the range of 2–300 min. Thus, in further experiments, a 10 min short-term stability was applied. Although any time within the interval could be also applicable.

### 3.8. The New Polymer Inclusion Membrane-Based Optical Sensor for the Determination of Fe(II) Ions

The components of the PIM (0.59 g CTA, 0.04 g SHPA, 4.9 mL DBP, and 38 mL DCM) are dissolved by means of an ultrasound generator (8 min of sonication pulses at 2 s intervals) to obtain a homogeneous mixture. The mixture is then placed in an 11.7 cm diameter glass Petri dish, allowing the DCM to evaporate into a dark fume cabinet for a curing time with room control temperature at 20 °C. The resultant PIM is sliced into portions of 2.37 cm × 0.98 cm in size and stored, in the absence of light, inside a desiccator until it is used as an optical sensor. The time lag between the synthesis of the PIM and its exposure to the metal solution without significant changes in the sensor response is at least 144 h.

Batch experiments consist in exposing the optical sensor to 20 mL of sample solution buffered with a 0.337 mol L^−1^ acetic acid/sodium acetate solution at pH 6 and a time of exposure of 90 min in 100 mL polypropylene containers. The containers are shaken at 300 rpm at a controlled temperature of 20 °C during the time of exposure. The sensor is then taken out from the container, rinsed with ultra-purified water, dried with absorbent paper, and placed onto a polyester film support for the UV-Vis measurements after 10 min. 

### 3.9. Analytical Performance of the Method

Under optimal conditions, and following the procedure described in Section 3.8, the limit of detection (LD) and quantification (LQ), the linear range, and the sensitivity were evaluated in both maximum absorption peaks (389 and 645 nm). The LD and LQ were determined as 3 σ/m and 10 σ/m, respectively, with σ being the standard deviation of the blank signal, and m being the slope of the linear calibration plot (*n* = 10). 

**Figure 6:**
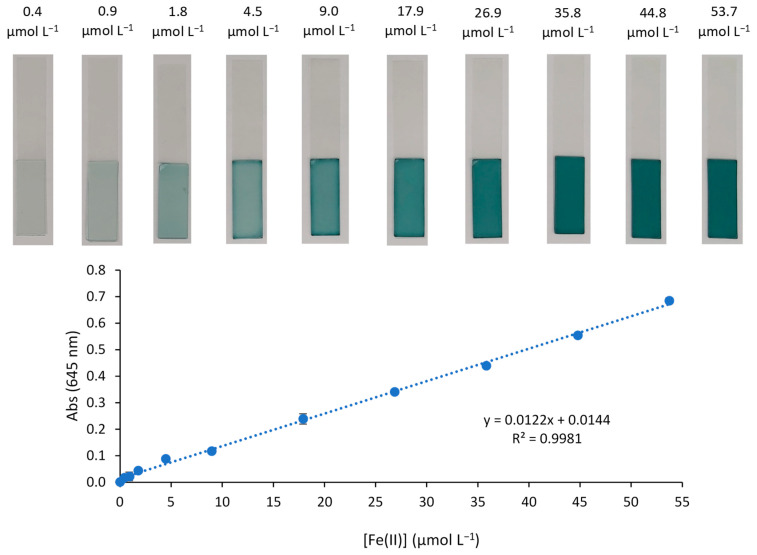
Optical sensor exposure to different concentrations of Fe(II) corresponding to the calibration curve (*n* = 2) with PIM pictures for each concentration.

For the 645 nm wavelength, the LD and LQ values were 0.48 and 1.59 µmol L^−1^ Fe(II), respectively. The linear range was evaluated using different solutions of Fe(II) with concentrations up to 54 µmol L^−1^. The response of the sensor to the Fe(II) concentration was linear fitted to the following equation (*n* = 10):

Abs (645 nm) = (0.0122 ± 0.0002) [Fe(II)] (µmol L^−1^) + (0.0144 ± 0.0046) (2)

obtaining a correlation coefficient of R^2^ = 0.9981 and a standard error of estimate of 0.011 (Figure 6). The precision of the method (at a confidence interval of 95%) was determined from eight replicate experiments using two different Fe(II) concentrations (17.9 and 45 µmol L^−1^), and was found to be 1.44% and 1.19%, with a relative standard deviation of 1.73% and 1.42%, respectively.

For the 389 nm wavelength, the LD and LQ values were 0.21 and 0.69 µmol L^−1^ Fe(II), respectively. The linear range was evaluated using different solutions of Fe(II) with concentrations up to 17.9 µmol L^−1^. The response of the sensor to the Fe(II) concentration was linear fitted to the following equation (*n* = 10):

Abs (389 nm) = (0.1558 ± 0.0045) [Fe(II)] (µmol L^−1^) + (0.1266 ± 0.0353) (3)

obtaining a correlation coefficient of R^2^ = 0.9958 and a standard error of estimate of 0.0725. 

Based on the results, the absorbance measured at 389 nm provide a sensitivity 12.8 times higher than the one obtained with measurements at 645 nm and lower LD and LQ values, but a narrow linear range due to detector saturation and, as mentioned in Section 3.1, subject to other metal complex interferences. Both wavelengths can be used in the determination of Fe(II) depending on the sensitivity needed and the interferences present in the samples. 

The selectivity of the sensor response (at 645 nm) was evaluated by studying the degree of interference found in the presence of different metal ions such as Ag(I), Bi(III), Cd(II), Co(II), Cr(III), Cu(II), Fe(III), K(I), Mg(I), Ni(II), Pb(II), and Zn(II), and anions such as chloride and fluoride. For that purpose, solutions containing 1:1 molar ratio of Fe(II):interfering ion, with Fe(II) concentrations of 17.9 µmol L^−1^, were obtained from Mohr’s salt.

**Figure 7:**
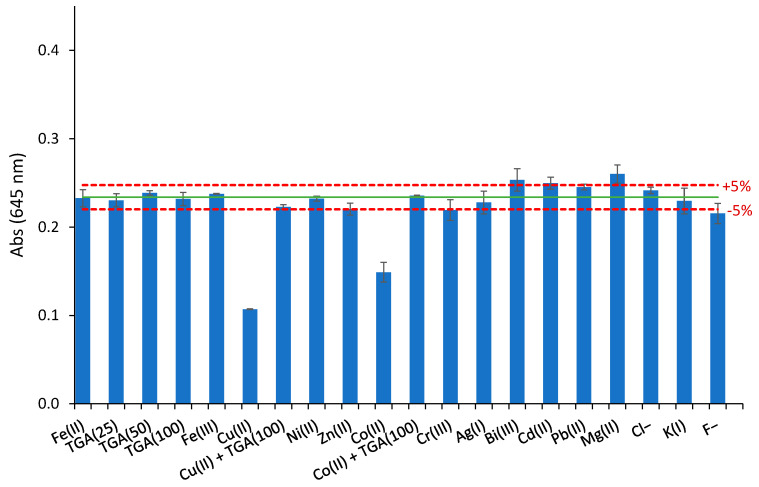
Interfering effect of different ions and thioglycolic acid (TGA) as a masking agent on the response of the sensor at a 1:1 molar ratio of Fe(II):interfering ion and different molar ratios of Fe(II):thioglycolic acid: 1:25 (TGA25), 1:50 (TGA50), and 1:100 (TGA100) ([Fe(II)] = 17.9 µmol L^−1^ for all solutions, *n* = 2). Green line corresponds to the absorbance value of the sensor exposed to 17.9 µmol L^−1^ Fe(II).

The results (Figure 7) indicated that most of the studied ions produced low interference or did not affect the Fe(II) response (5% tolerance limit), except Cu(II) and Co(II). Fe(II) in samples with the presence of these two elements could be analyzed using thioglycolic acid (TGA) as masking agent [58]. Thus, experiments to study the masking effect of this reagent were carried out using Fe(II) solutions containing 1:25, 1:50, and 1:100 molar ratio of Fe(II):thioglycolic acid. The results (Figure 7) showed that the use of this masking agent did not affect the sensor response to Fe(II), and the interferences of Cu(II) and Co(II) can be eliminated by using thioglycolic acid.

### 3.10. Speciation Analysis of Fe(II)/Fe(III) with the New Proposed Optical Sensor

From the interference study, it can be concluded that the presence of Fe(III) in the sample did not affect the Fe(II) response. To study the application of the proposed optical sensor for the speciation of Fe(II)/Fe(III), the analysis of three synthetic metal solutions was carried out: (1) solution containing 17.9 µmol L^−1^ Fe(III); (2) solution containing 17.9 µmol L^−1^ Fe(III) and 17.9 µmol L^−1^ Fe(II); and (3) solution containing 17.9 µmol L^−1^ Fe(III) with 0.0289 mol L^−1^ ascorbic acid. The results obtained are shown in Table 3. 

**Figure 8:**
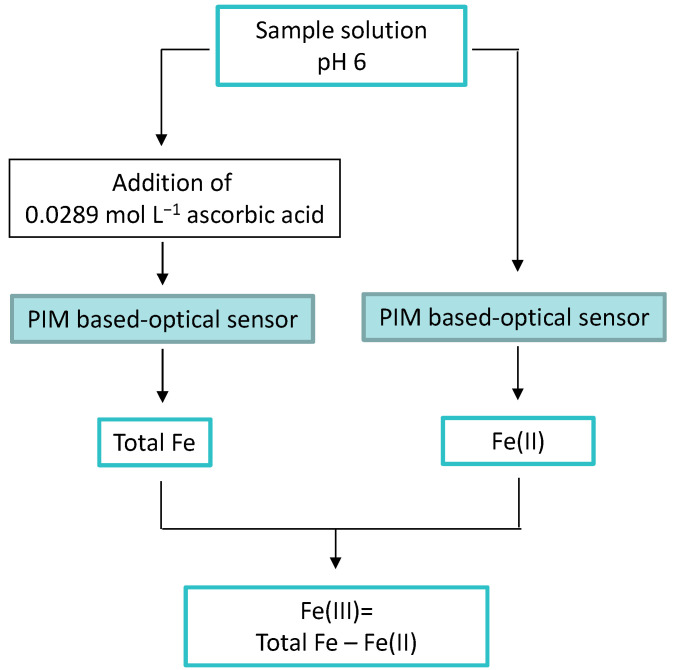
Speciation scheme of Fe(II)/Fe(III) in samples applying the proposed PIM-based optical sensor.

Based on these results, a scheme for the speciation analysis of iron can be proposed (Figure 8). The use of ascorbic acid in the samples allowed the evaluation of total Fe (as Fe(III) + Fe(II)), and the Fe(III) concentration could be calculated as the difference between the total Fe and the Fe(II), measured without the addition of ascorbic acid.

### 3.11. Analytical Applications

The accuracy of the method was studied by sensing analysis of Fe(II) ions in black olive brines, Tardyferon (iron supplement) and a food additive (E579). The results were compared to the values obtained by the ortho-phenanthroline method, using a Student’s *t*-test (95% confidence). There were no significant differences in the quantitative determination of Fe(II) between the two methods (Table 4). As a consequence, the designed optical sensor can be successfully applied in the determination of Fe(II) ions in real samples, providing accurate results. 

The applicability of the proposed speciation scheme was also evaluated by the determination of Fe in the fertilizer sample. The results obtained were 5.44% of total Fe, with 0.01% being of Fe(II), and the percentage of Fe(III) being 5.43%. The total Fe value is in accordance with the data provided by the manufacturer.

It is worth mentioning that the literature related to sensors of iron mainly focuses on the detection of Fe(III) ions or total Fe. Therefore, it has been observed that designs of polymeric optical sensors to determine Fe(II) are limited. Some of them have been compared in Table 5.

The range of pH value of the feed solutions was from 2 to 6. Green methodologies should use moderate or non-acidic solutions as those proposed in this paper, which work with feed solutions at pH 6, such as the 2-(2-pyridyl) imidazole probe [48]. However, this latter sensor offered a high LOD, and few analytical parameters were evaluated.

Wide ranges of linearity were found for some sensing membranes, but their limits of detection were higher than the one provided by the proposed sensor (for ferrozine-PVC sensor with LOD of 4.5 µmol L^−1^ [47] and for 1,10-phenanthroline-alginate/pectin sensor with LOD of 7.99 µmol L^−1^ [45]). In other studies with wide ranges, the detection limit was not evaluated [44] or was similar (for ferrozine-D4 hydrogel with LOD of 0.34 µmol L^−1^) to that of this work (LOD of 0.48 µmol L^−1^), but the RSD was higher, and interferences were not studied [29]. 

As can be seen, some studies indicated that the sensor membranes based on the immobilisation of ferrozine were applied in non-equilibrium steady state, providing higher RSD values of up to 12.12%, especially when short sensing times were applied [29,46,47]. A similar behaviour was shown by the 1,10-phenanthroline-poly(acrylamide) sensor for which the equilibration time evaluated was 2 h but the exposure time of the sensor to Fe(II) was only 15 min, offering a high RSD value of 8 [42]. 

The sensor used in this work outstands for its sensitivity (0.0122 and 0.1558 absorbance units/(µmol L^−1^) at 645 nm and 389 nm, respectively), and was only surpassed by the ferrozine-D4 hydrogel sensor (0.2595 absorbance units/(µmol L^−1^) [46]), which showed a high RSD (7.6%). A similar sensitivity value to the one obtained in this work was found for silica sol-gel film (0.117 absorbance units/µmol L^−1^), which offered a low LOD (0.03 µmol L^−1^), but yielded a narrow linear range (0.1–2.1 µmol L^−1^) [43].

Co(II) and Cu(II) ions were the ions that most frequently interfered in Fe(II) sensing. The common Fe(II):metal ion ratio studied was 1:1, although most real samples contain a higher abundance of iron as a minor component than other transition metal ions (trace components). For this reason, the interferences of the ferrozine-PVC sensor [47] were investigated using ratios of 100:1 or 1000:1, which did not result in any interference. In the present work, these interferences were successfully avoided by using the masking agent TGA even in a 1:1 ratio.

Few sensors allowed Fe speciation, which raises great interest in iron analysis. Thus, the silica sol-gel sensor [43] and the SHPA-PVC sensor proposed in this work could be applied to determine Fe(II) and total Fe, and therefore, Fe(III) by difference.

## 4. Conclusions

In this study, a green optical sensor for the selective determination of Fe and its speciation was designed and applied in real samples. The sensor was based on the immobilization of the chromogenic reagent picolinaldehyde salicyloylhydrazone (SHPA) in a polymer inclusion membrane. This reagent, prepared by a mechanochemical process using minimal amounts of solvents, is capable of forming a colored complex with Fe(II) ions, allowing the spectrophotometric measurements. The composition of the polymer inclusion membrane was optimized with a sequential simplex method and the proposed sensor was characterized in detail. The new green optical sensor offers good reproducibility, selectivity, and optical properties, allowing the design of a Fe(II)/Fe(III) speciation scheme. The proposed methodology was successfully applied to the determination of iron in food preservatives, food additives, a dietary supplement, and, additionally, the Fe speciation scheme was successfully applied to an agricultural fertilizer.

## Figures and Tables

**Table 1:** Response at 389 and 645 nm of the optode to 90 µmol L^−1^ Fe(II) using different compositions of PIM with SHPA as the reagent (0.02 g) at different pH values (*n* = 2).

Membrane	Polymer	Plasticizer	Additive	Solvent	pH	Abs 389 nm		Abs 645 nm	
						Mean	SD	Mean	SD
1	PVC	TBP	-	THF	4.6	0.2730	0.0343	0.0256	0.0048
	(2.5014 g)	(3 mL)		(19.1 mL)					
2	PVC	NPOE	NaTPB	THF	4.6	0.0268	0.1233	0.0212	0.0343
	(0.8013 g)	(1.54 mL)	(0.0205 g)	(20 mL)					
3	CTA	DBP	-	DCM	4.6	0.4986	0.0036	0.0500	0.0050
	(1.0024 g)	(4 mL)		(35 mL)					
4	CTA	DBP	-	DCM	2.5	0.0003	0.0096	-	-
	(1.0024 g)	(4 mL)		(35 mL)					
5	CTA	DBP	-	DCM	9	0.1055	0.0153	0.0124	0.0103
	(1.0024 g)	(4 mL)		(35 mL)					

**Table 2:** Simplex optimization of the variables for the synthesis of the optical sensor (Conditions: metal solution containing 90 µmol L^−1^ Fe(II), buffered by 0.337 mol L^−1^ acetic acid/sodium acetate solution at pH 4.6; exposure time of 60 min; shaken at 300 rpm at a controlled temperature of 20 °C).

Vertex	CTA (g)	SHPA (g)	DBP (mL)	DCM (mL)	Abs (abs. Unit)	
					Mean	SD
1 *	1	0.02	4	35	0.0528	0.0016
2 *	2	0.02	4	35	0.0128	0.0039
3 *	1	0.06	4	35	0.0892	0.0088
4 *	1	0.02	8	35	0.0263	0.0001
5 *	1	0.02	4	70	0.000	0.000
6	1.5	0.04	6	0.05	Rejected	Rejected
7	1.1	0.03	4.5	52	0.0132	0.0062
8	0.06	0.04	6.2	44	Rejected	Rejected
9	1.5	0.03	4.6	37	0.0398	0.0027
10	1.1	0.04	5.8	19	Rejected	Rejected
11	1.1	0.03	4.8	44	0.0664	0.0016
12	1.3	0.05	0.7	41	Rejected	Rejected
13	1.1	0.03	6.2	36	0.0518	0.0025
14	0.59	0.04	4.9	38	0.0891	0.0026
15	0.78	0.05	2.7	40	0.0443	0.0007
16	1	0.03	5.3	37	0.0528	0.0007
17	0.86	0.06	5.5	42	0.0607	0.0012

* Initial simplex.

**Table 3:** Speciation analysis of Fe(II)/Fe(III) with the new proposed optical sensor under optimal conditions.

Added Metal Concentration (µmol L^−1^)		Presence of Ascorbic Acid	Determined Fe (II) Concentration (µmol L^−1^)
Fe(III)	Fe (II)		
17.9	0	No	<LD
17.9	17.9	No	18.30 ± 0.04
17.9	0	Yes	18.4 ± 1.6

**Table 4:** Comparison of Fe(II) determination in real samples by the proposed optical sensor and the ortho-phenanthroline method (t_tab_ (95%) = 4.303).

Sample	Fe(II) ± SD (µmol L^−1^)		t_calc_
	Optical Sensor	Ortho-Phenanthroline Method	
Black olive brine (Karina)	13.25 ± 0.35	12.98 ± 0.24	0.883
Black olive brine (Ifa Eliges)	4.25 ± 0.43	4.17 ± 0.13	0.231
Food additive E579	21.61 ± 0.35	20.94 ± 0.08	2.661
Tardyferon	20.73 ± 1.47	20.79 ± 0.27	0.062

**Table 5:** Comparison of the proposed Fe(II) optical sensor with other reported methods.

Immobilized Reagent	Polymer Matrix	pH of Sol.	V Sample (mL)	Linear Range (µmol L^−1^)	Sensitivity * (Abs. Unit/(µmol L^−1^))	LOD (µmol L^−1^)	RSD (%) ([Fe(II)] (µmol L^−1^))	Metal Interferences (Ratio Fe(II):Metal)	Application to Real Samples	Remark	Ref.
Ferrozine	D4 polyurethane hydrogel membrane	2.5	100	0.73–5.37	0.0195	0.21	4.9 (2.7 µmol L^−1^)	Cu(II) (1:1.2) Co(II) (1:2)	Waters and wines	Measures in non-equilibrium steady state; interference of wine colorants.	[46]
				0.07–0.90	0.2595	0.02	7.6 (0.5 µmol L^−1^)				
Ferrozine	D4 polyurethane hydrogel membrane	4.0	10	17.9–179	0.0011	0.34	12.12 (35.8 µmol L^−1^) 4.77 (89.6 µmol L^−1^)	Not studied	n.m.	Measures in non-equilibrium steady state.	[29]
Ferrozine	poly(vinyl alcohol) membrane	5.5	n.m.	5–200	n.m.	4.5	4.6 (50 µmol L^−1^) 2.3 (150 µmol L^−1^)	None Zn(II), Cu(II), Pb(II), Al(III), Fe(III) (100:1) Co(II), Ni(II) (1000:1)	Seawater and marine sediment	Measures in non-equilibrium steady state.	[47]
2-(2-pyridyl) imidazole	Nanofibers of poly(vinyl benzyl chloride)	6	n.m.	n.m.	n.m.	35.8	n.m.	Ni(II), Cu(II), Co(II) (1:1)	n.m. for sensing probe	Probe analytical parameters not studied.	[48]
1,10-phenan-throline	Poly(acrylamide) grafted poly(propylene) membrane	3	25	0.36–35.8	0.0082	0.36	8 (n.m.)	Co(II) (n.m.)	Ground waters and fruit juice	Equilibrium steady state at 2 h, but sensing at 15 min. Visual/instru-mental detection.	[42]
2,4,6-tri(2-pyridyl)-s-triazine	Silica sol-gel film	3	5	0.09–2.1	0.117	0.03	3.5 (0.17 µmol L^−1^) 1.27 (1.61 µmol L^−1^)	None	Tap, well and river water	Fe speciation	[43]
N,N′- Ethylene *bis*(salicyl-imine)	Poly(vinyl chloride) membrane	n.m.	n.m.	1–1000	n.m.	n.m.	n.m.	n.m.	Tap and mineral waters	Few analytical parameters studied.	[44]
1,10-phenan-throline	Alginate/pectin film	2	2	0–179	0.0008	7.99	0.7–3.13 (n.m.)	Fe(III) (1:1)	Spiked tap water	Visual/instru-mental detection.	[45]
Picolin-aldehyde salicyloyl-hydrazone	Cellulose triacetate membrane	6	20	0.48–54 (645 nm) 0.21–18 (389 nm)	0.0122 (645 nm) 0.1558 (389 nm)	0.48 (645 nm) 0.21 (389 nm)	1.73 (17.9 µmol L^−1^) 1.42 (45 µmol L^−1^)	None (1:1)	Black olive brines, dietary supplement, food additive, fertilizer	Measures in equilibrium steady state. Fe speciation	This study

* Sensitivity defined as the slope of the calibration curve [59]; n.m.: not mentioned.

## Data Availability

Data is contained within the article.

## Appendix A

The experimental setup is shown in Figure A1.

To study the effect of the ascorbic acid concentration in the metal solution, the absorbance was evaluated after exposure of the PIM to Fe(III) reduced to Fe(II) with different concentrations of ascorbic acid (0, 0.0045, 0.0134, 0.0289, and 0.0403 mol L^−1^), buffered by 0.337 mol L^−1^ acetic acid/sodium acetate solution at pH 6 for 90 min at 300 rpm. The results showed (Figure A2) that the response was steady between 0.0134 and 0.0403. The maximum absorbance was obtained at 0.0289 mol L^−1^.

The UV-Vis absorbance spectrum of the SHPA-Fe(II) complex is shown in Figure A3.
